# Experimental Investigations on Subsequent Yield Surface of Pure Copper by Single-Sample and Multi-Sample Methods under Various Pre-Deformation

**DOI:** 10.3390/ma11020277

**Published:** 2018-02-10

**Authors:** Gui-Long Liu, Shi-Hong Huang, Che-Si Shi, Bin Zeng, Ke-Shi Zhang, Xian-Ci Zhong

**Affiliations:** 1 Key Lab of Disaster Prevent and Structural Safety, Guangxi Key Lab Disaster Prevent and Engineering Safety, College of Civil Engineering and Architecture, Guangxi University; Nanning 530004, China; guilongliu@163.com (G.-L.L.); hshhshhsh1234@126.com (S.-H.H.); shichesi@126.com (C.-S.S.); zengbin379@163.com (B.Z.); 2 College of Mathematics and Information Science, Guangxi University, Nanning 530004, China; xczhong@gxu.edu.cn

**Keywords:** pure copper, subsequent yield measurement, pre-strain, concave

## Abstract

Using copper thin-walled tubular specimens, the subsequent yield surfaces under pre-tension, pre-torsion and pre-combined tension-torsion are measured, where the single-sample and multi-sample methods are applied respectively to determine the yield stresses at specified offset strain. The rule and characteristics of the evolution of the subsequent yield surface are investigated. Under the conditions of different pre-strains, the influence of test point number, test sequence and specified offset strain on the measurement of subsequent yield surface and the concave phenomenon for measured yield surface are studied. Moreover, the feasibility and validity of the two methods are compared. The main conclusions are drawn as follows: (1) For the single or multi-sample method, the measured subsequent yield surfaces are remarkably different from cylindrical yield surfaces proposed by the classical plasticity theory; (2) there are apparent differences between the test results from the two kinds of methods: the multi-sample method is not influenced by the number of test points, test order and the cumulative effect of residual plastic strain resulting from the other test point, while those are very influential in the single-sample method; and (3) the measured subsequent yield surface may appear concave, which can be transformed to convex for single-sample method by changing the test sequence. However, for the multiple-sample method, the concave phenomenon will disappear when a larger offset strain is specified.

## 1. Introduction

Metal material yield and subsequent yield have been the focus of many scholars’ studies [1,2,3]. The subsequent yield surface is used not only to describe the limit of the elastic deformation of a material, but also to determine the spatial direction of plastic deformation (the normal flow rule). The reasonable description of the subsequent yield surface is an important basis of plastic theory. However, it is found that the position and shape of the subsequent yield surface of the material in the stress space are anisotropic in the loading process, due to the plastic deformation and the Bauschinger effect [4,5,6]. The yield surface will significantly deviate from the initial state and its expansion is inhomogeneous. Near the pre-loading point, the curvature of the yield surface locus becomes larger and shows a “sharp corner” phenomenon, after the preloading along a certain direction; and in the opposite direction, curvature becomes smaller and more flattened [7,8,9]. The change of the yield surface on the normal of the preloading direction will occur, which is called the cross effect [10,11,12,13,14,15]. The shape of the measured yield surface becomes more and more different from the cylindrical surface described by the classical plasticity theory with the increase of plastic deformation. 

Most of the previous experimental investigations on the evolution of the yield surface have been carried out by the probe methods using a single specimen, such as Khan et al. [13], Khan et al. [14], Phillips and Tang [16], and Sung et al. [17]. Since the single-sample method is used to measure multiple yield points through a polygonal path, additional plastic deformation accumulation will occur. Strictly speaking, the measured yield points do not belong to the same yield surface. Therefore, the yield must be determined by a very small specified offset strain or a linear deviated value (Helling et al. [7] used 5 με offset strain, Ellis et al. [18] used 10 με offset strain, Hu et al. [19] compared the difference of measured yield surfaces by using 10 and 20 με offset strains to define the yield). Even so, it is hard to confirm the feasibility of the measured yield surface. However, the multi-sample method is different as it is free of additional plastic deformation accumulation. But the results may be affected by the dispersion caused by different specimens. From the existing literature, there is still less experimental study on the subsequent yield measured by multi-sample method (Hu et al. [19], Stout et al. [20], Khan and Wang [21], Wu and Hong [22], and Hu et al. [23]).

Bishop and Hill [24] pointed out that if a single crystal obeys the Schmidt law, it would result in the yield surface being convex. Furthermore, based on Drucker’s postulate, the convexity of the yield surface can also be proved. However, the experiment adopting the thin-walled tubular sample under axial-torsion loading may lead to the result that the subsequent yield surface is concave [19].

To improve the existing theory, it is necessary to make clear the differences of subsequent yield surface between that actually tested and that suggested by classical plasticity theory. In this paper, the evolution of the yield surface of a pure copper after different pre-deformation is studied by using both the single-sample method and multi-sample method. On the one hand, we want to provide additional examples for the observation of the evolution of the yield surface; on the other hand, we aim to explore the rationality and limitations of the two experimental methods.

## 2. Experiments

**Figure 1:**
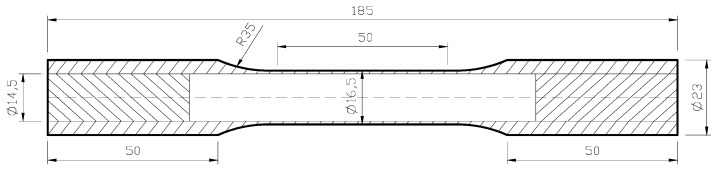
The geometry size of the specimen (unit: mm).

The tested materials are bars made of industrial pure copper (T2) with an original diameter of 25 mm. Its chemical composition in weight % is given in Table 1. In order to eliminate the hardening effect caused by the forming process, the material is treated by stress-relieving annealing. The specimens are thin-walled and tubular, and the geometry of the specimen is shown in Figure 1. By using numerical control machining technology, the inner and outer surfaces of the gauge section are polished. In order to ensure reliable clamping, both ends of each tubular specimen are filled with additional steel inserts.

Experiments are carried out on an MTS809 axial-torsion servo hydraulic fatigue testing machine (MTS Systems Corporation, Minneapolis, MN, USA). The axial-torsion extensometer MTS632.80F-04 (MTS Systems Corporation, Minneapolis, MN, USA) with the gauge length of 25 mm is fixed on the middle of the specimen to measure the axial and torsion deformations. The basic mechanical parameters of materials measured through tests are presented in Table 2.

**Figure 2:**
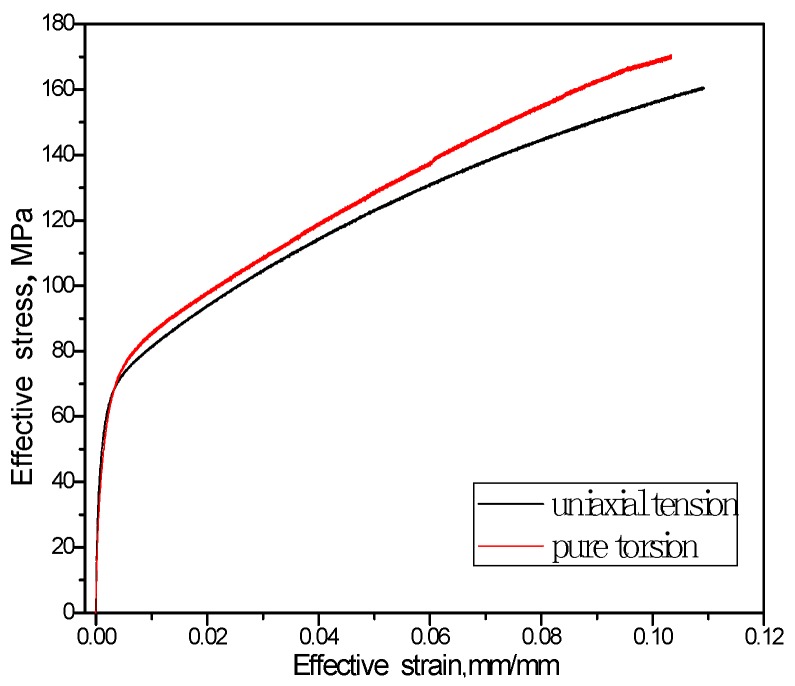
Equivalent stress-strain curve of T2 pure copper under monotonic loading.

Equivalent stress–equivalent strain curves of the uniaxial tension and pure torsion test of T2 pure copper are shown in Figure 2. Both of them are similar and exhibit no obvious macroscopic yield plateau.

The material’s subsequent yield point, i.e., the elastic limit reached after the material has undergone plastic deformation, and its determination and identification can be achieved by measuring the linear deviation of the loading curve or the offset strain due to unload leaded residual deformation. The latter is named the offset strain method, by which the tested results are stable, meaning that this method is feasible and credible. When the material is loaded from the specified start point within the elastic range and unloaded to the point, the strain will revert to the previous state without translation (deviation). However, when the elastic range is exceeded, the strain measured after unloading will be shifted, and the magnitude of the strain will be related to the extent beyond the range. Therefore, the measured offset strain can be used as a criterion for judging the yield of the material. The smaller the offset strain judging the yield of the material, the closer it is to the description given by the theory. For the analysis of plasticity, the constitutive model has a certain degree of empirical expression and has been simplified. At the same time, since the small jitter of the test machine and the viscosity of the material, it is of little significance to specify a small offset strain, and the actual test is also difficult to achieve. Therefore, the offset strain should be specified according to the requirements and the stable identification of the test machine. The measurement of linear deviation is very difficult to operate in situations where the deviation is very small. One issue is whether the material is strictly linear in the elastic range, and secondly, all kinds of factors will affect the linear deviation measurement of the loading response in the case of high precision requirements.

### 2.1. Subsequent Yield Surfaces Tested by Using Single- and Multi-Sample Methods 

The yield points of different loading directions (different axial stress-shear stress ratio) are measured by one specimen in turn through the specified offset strain using single-sample method. As for the multi-sample method, one specimen is only measured in the yield points of one specified loading direction (different specified offset strain can be chosen). A yield surface is determined by different yield points tested by multiple specimens along different loading directions. In this paper, the yield is determined according to the target value of the offset strain tested by the equivalent plastic strain. It can reflect the influence of normal and shear deformation components.

**Figure 3:**
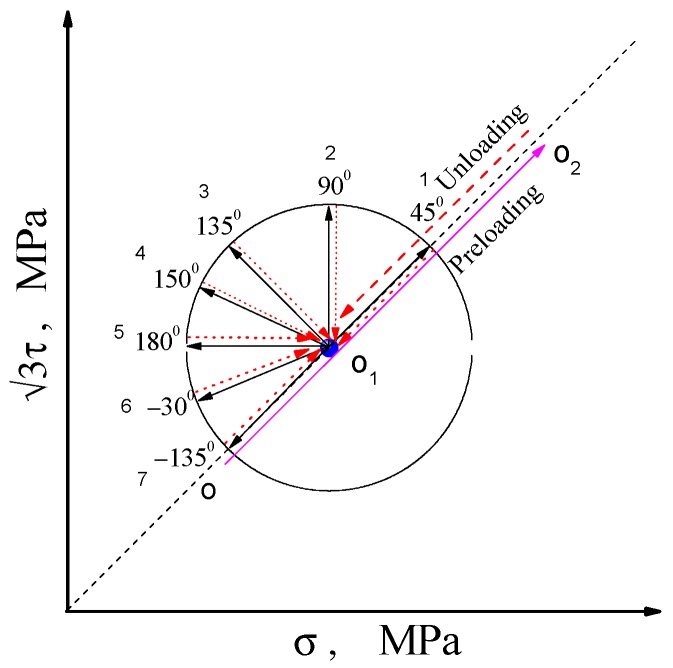
Schematics of probing path for subsequent yield surface.

In Figure 3, the method of testing yield surface is implemented by axial stress–shear stress loading. The point O in Figure 3 represents the initial point of the material without plastic deformation. From this point, loading along the O–O_2_ direction to the point O_2_ in the plastic region (called pre-deformation point), then unloading back to the specified point O_1_ in the elastic region, it is generally considered that there are residual stress, elastic residual strain and plastic residual strain at this point of the material. In the graphical plane, the loading from this point to any direction will undergo an elastic process and then enter a plastic deformation. Accordingly, the yield point of the corresponding path can be measured according to the specified offset strain. Seven optional directions for starting from the point O_1_ and returning to the point O_1_ are shown in Figure 3. 

**Figure 4:**
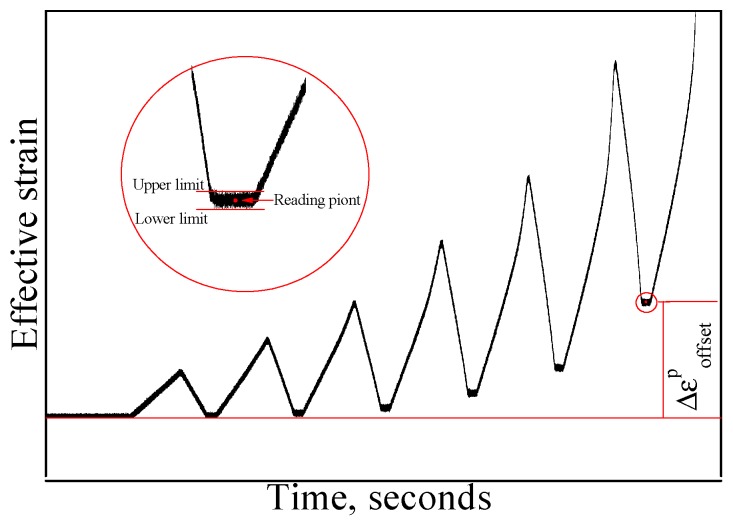
Schematic diagram of offset strain test through gradual reloading and unloading.

We use one specimen to test the yield points in seven directions in turn for the single-sample method. However, for the multiple-sample method, one specimen is required in each direction to test the yield point. For both methods, the residual plastic strain accumulated during loading and unloading gradually in each direction is measured to compare the specified offset strain. The corresponding peak stress is the yield stress when the cumulative plastic strain reaches the target value of the offset strain, as showed in Figure 4. 

**Figure 5:**
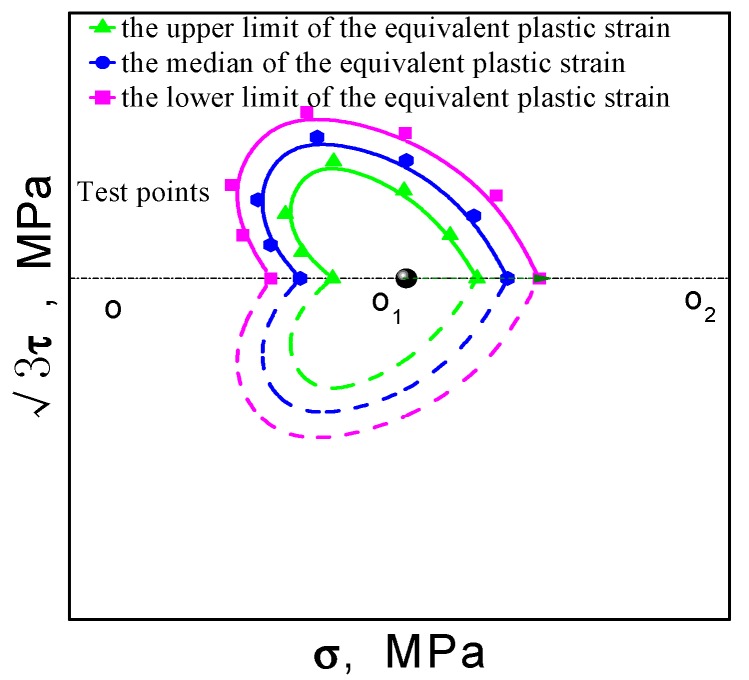
Schematic diagram of the tested yield surface distributed within a range.

The measured value of the equivalent plastic strain is generally affected by the noise of the measurement, as shown in Figure 4, and the equivalent plasticity strain is not a determined value, but beats within a certain strain range. Therefore, the size of the tested yield surface is also distributed within a certain range, as shown in Figure 5. In this paper, the intermediate value of the equivalent plastic strain is used to determine the offset strain.

From the above statements, it is seen that when we test a yield point in one loading direction by the single-sample method, the plastic strain of the specimen will make a slight change. Thus, the tested yield points actually correspond to the different yield surfaces under diverse plastic deformation. Additional experiments are needed to prove whether their differences are large or small.

To get a reasonable result, the target value of the offset strain by using the single-sample method must be small: for example, 20 με. This has been difficult when using the existing test machines, because errors will be caused by small jitters during the loading process. In this process, we consider the material to be in the yield state when the error between the accumulated equivalent plastic strain (see Figure 4) and the target value of offset strain is less than 2 με. This value is very small and can hardly be distinguished. When the multi-sample method is applied, one specimen is loaded only in a specified direction and subjected to yield test with different values of specified offset strain, hence a series of yield surfaces under different offset strain values can be measured. The yield points in different directions are obtained by respective specimens loading along different directions. The most critical point is that the single-sample method is subject to the influence of the cumulative plastic strain caused by previous loading along different directions, while the multi-sample method will not be affected.

### 2.2. Data and Parameter Calculation of Yield Surface Test

Axial force *F*, torque *T*, elongation at gauge section ΔL and torsion angle are recorded in the output data of the test. The nominal axial stress σ and shear stress τ nominal axial strain ε and shear strain γ of the thin-walled tubular specimen is calculated as follows:

(1) $$\sigma =\frac{4F}{\pi (D^{2}-d^{2})},\tau =\frac{16T}{\pi (D^{2}-d^{2})(D+d)},\varepsilon =\frac{\Delta L}{L},\gamma =\frac{\bar{R}\cdot \theta }{L}$$

where *D* is the outer diameter and *d* is the internal diameter of the thin-walled tubular specimens in the gauge section; $\bar{R}$ and *L* are the mean radius and initial length on the gauge section. The equivalent stress $\sigma_{eq}$ and equivalent residual plastic strain $\Delta \varepsilon_{eq}^{p}$ relative to the unloading point by the following formula:

(2) $$\sigma_{eq}=\sqrt{\sigma^{2}+3\tau^{2}},\Delta \varepsilon_{eq}^{p}=\sqrt{{\left(\Delta \varepsilon^{p}\right)}^{2}+\frac{{\left(\Delta \gamma^{p}\right)}^{2}}{3}},\Delta \varepsilon^{p}=\Delta \varepsilon -\frac{\Delta \sigma }{E},\Delta \gamma^{p}=\Delta \gamma -\frac{\Delta \tau }{G}$$

where *E* and *G* are the elastic modulus and shear modulus of the material, respectively, the above stress and strain increments are calculated with reference to the unloading point defined by the following expression:

(3) $$\Delta \varepsilon =\varepsilon -\varepsilon_{O_{1}},\Delta \gamma =\gamma -\gamma_{O_{1}},\Delta \sigma =\sigma -\sigma_{O_{1}},\Delta \tau =\tau -\tau_{O_{1}}$$

For the multi-sample method, $\varepsilon_{o1}$ and $\gamma_{o1}$ are invariant, while for the single-sample method, they are changed at each yield point (although the amount of change may be small). However, regardless of single or multiple-sample method, $\sigma_{o1}$ and are unchanged at the unloading point.

In the process of testing, when the cumulative equivalent residual plastic strain $\Delta \varepsilon_{eq}^{p}$ is equal to the specified offset strain (using $\Delta \varepsilon_{offset}^{\mathrm{residual}}$ to represent) during the successive loading process, the axial stresses and shear stresses can be computed by the Equations (1) and (2). Thus, the yield surface (curve) is depicted by a series of yield points in the $\sigma \sim \sqrt{3\tau }$ plane (space).

## 3. Comparison of the Results of Single- and Multi-Sample Methods

**Figure 6:**
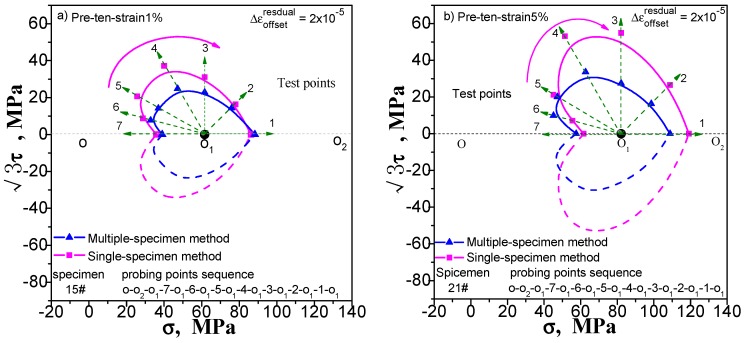
Comparison of subsequent yield surface measured by single-sample and multi-sample method: (**a**) probing after pre-tension at 1%; (**b**) probing after pre tension at 5%.

**Figure 7:**
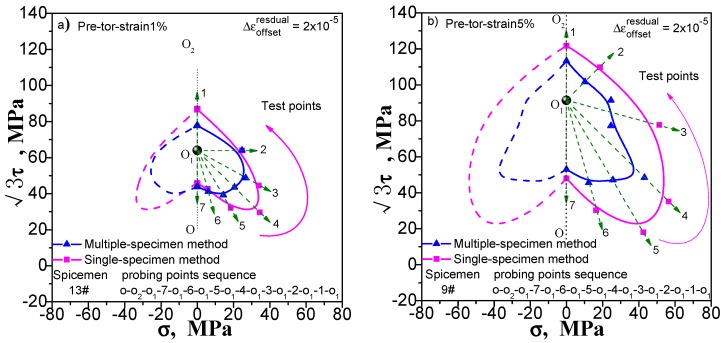
Comparison of subsequent yield surface measured by single-sample and multi-sample method: (**a**) probing after pre-torsion at 1%; (**b**) probing after pre torsion at 5%.

**Figure 8:**
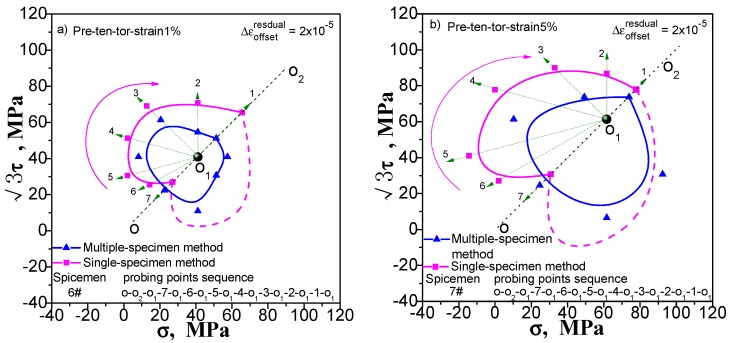
Comparison of subsequent yield surface measured by single-sample and multi-sample method: (**a**) probing after combined pre-tension–torsion at 1%; (**b**) probing after combined-pre tension–torsion at 5%.

The results of the single-sample method and the multi-sample method are compared to verify the effectiveness of the two methods. For comparison, the single- and multi-sample methods are used to adopt 20 με as the offset strain (or the target value of the offset strain) to determine the subsequent yield point under different pre-deformation. We give these contrast diagrams of the subsequent yield surface of pure copper (T2) measured by the single-sample and the multi-sample methods undergoing pre-tension at 1% and 5% (Figure 6), pre-torsion at 1% and 5% (Figure 7), combined pre tension-torsion at 1% and 5% (Figure 8). There are several explanations. (1) The test of each specimen by multi-sample method is independent and the test result of each yield point is not affected by the order of test points. The single-sample method has the sequence problem; (2) As can be seen from Figure 6, Figure 7 and Figure 8, the order of measuring points of a single sample method is based on reciprocal from 7 to 1. The dashed lines from the point O_1_ indicate the addition and unloading direction of the test yield stress in these diagrams; (3) The pre-deformation affects the symmetry of the yield surface along the directions of pre-deformation with respect to the opposite. However, it does not affect or little affects the symmetry of the yield surface in the directions with respect to both sides, and according to this hypothesis, the yield surface of the other side can be described by symmetry when the yield surface is measured.

One can see from Figure 6, Figure 7 and Figure 8 that (1) the shape of the subsequent yield surfaces measured by the two methods is similar. With the same method, the size of the yield surface is found to increase with the increase of pre-deformation. The overall position of the yield surface is found to shift to the right. It is shown that the subsequent yield surface will expand and move with the increase of preloading plastic deformation; (2) In the same case of pre-deformation, the location of the first measurement yield point (i.e., the measurement point of the serial number 7) determined by both the multiple-sample method and single-sample method is very close. However, the difference of followed measured position of the yield point is obviously increased. This phenomenon makes the size of the yield surface measured by single-sample method much larger than that measured by multiple-sample method and caused by the cumulative plastic strain of the single-sample method; (3) Either way, the measured yield surfaces appear as a “sharp corner” feature in the preload direction; (4) All the yield surfaces measured by single-sample method appear as a “concave” feature in the opposite direction of the preloading; although the “concave” feature of the subsequent yield surface measured by the multi-sample method is visible, it is not obvious; (5) From the measured yield surface results, one can see that the yield points measured by single-sample method are much smoother than that measured by multi-sample method. This phenomenon should be caused by the difference between specimens when multi-sample method is applied; (6) The locus of the succeeding yield surface measured either by single-sample or multiple-sample method is not consistent with the cylindrical yield surface proposed by the classical plastic theory (at present, the metal plasticity model provided by the common commercial software has adopted this hypothesis); (7) In the case of combined pre-tension–torsion (Figure 8), the yield points in different directions are measured by the multi-sample method around the unloading point, and the symmetry of the figure is not very good (further discussion will be given in Section 5.1).

The above results show that (1) the test results of the two methods are quite different, and their feasibility need to be further discussed; (2) in the investigation of the measured yield surface, all single-sample method test results and a few multiple-sample method test results may appear the “concave” feature, which is contrary to the classical plastic theory, and the reason for this needs to be given a reasonable explanation.

## 4. The Rationality of the Yield Surface Tested by Single-Sample Method

By definition, each point on the yield surface is not associated with the other points. In other words, each point is independent of other points. The multi-sample method does not violate this principle. The single-sample method needs to pass the multi spot test path. Because plastic deformation will increase at each measurement point, the follow-up test point result will certainly be affected by the preceding test point number and the path.

### 4.1. Differences in Test Results Caused by Different Test Points

**Figure 9:**
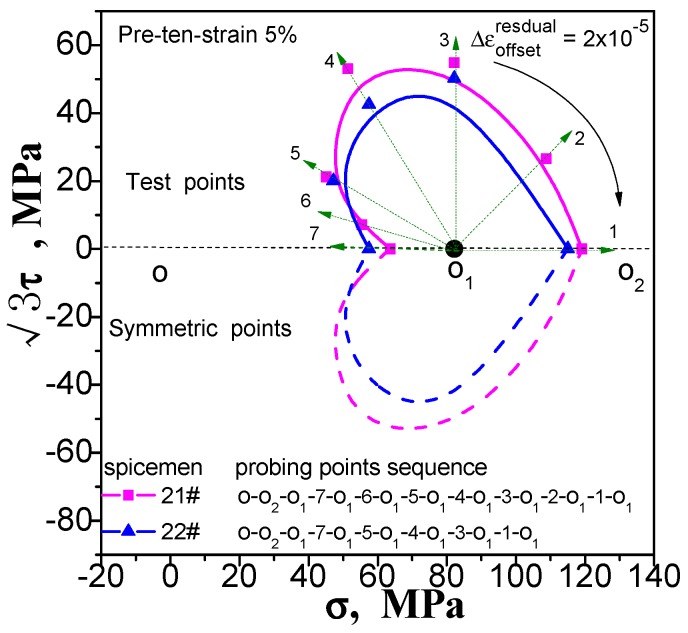
Influence of test point number on subsequent yield surface measured by single-sample method for the case of probing after pre-torsion at 5%.

**Figure 10:**
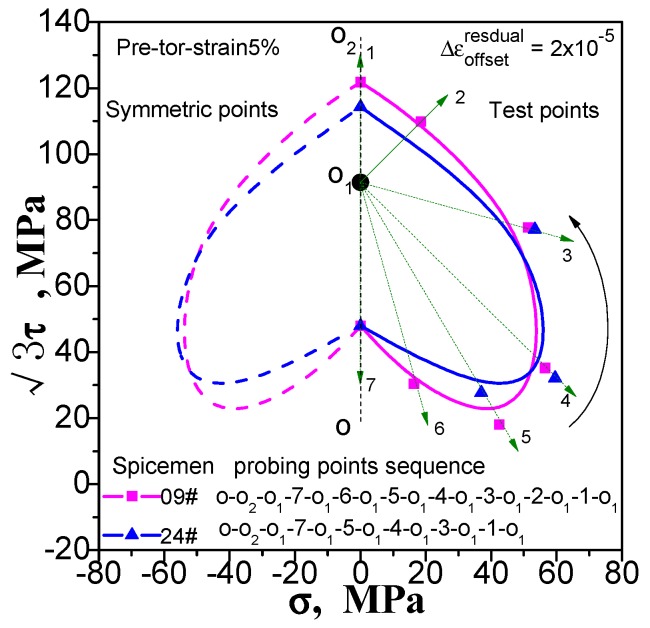
Influence of test point number on subsequent yield surface measured by single-sample method for the case of probing after pre-torsion at 5%.

**Figure 11:**
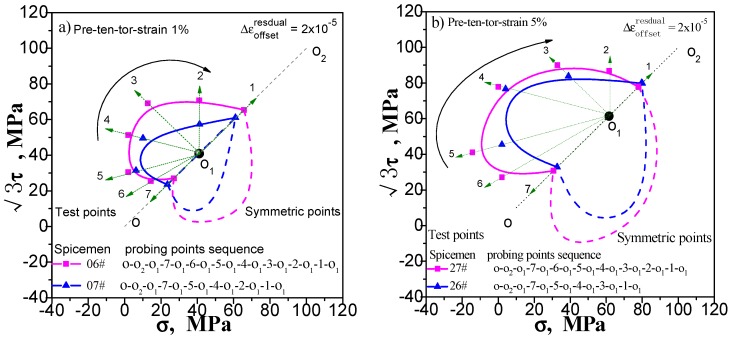
Influence of test point number on subsequent yield surface measured by single-sample method: (**a**) probing after combined pre-tension–torsion at 1%; (**b**) probing after combined pre-tension–torsion at 5%.

Enough measuring points can accurately describe the yield surface. This is not a problem for the multi-sample method, except that the cost of the test has been improved. However, this situation will have an impact on the results measured by single-sample method. If we increase the number of test point, the results of the following points will be affected by the accumulated residual strain caused by the previously test procedure. We still take the yield surface tests undergoing pre-tension at 1% and 5%, pre-torsion at 1% and 5%, and combined pre-tension–torsion at 1% and 5%. As an example, for each case, two specimens are used to test the subsequent yield surface by a single-sample method. To facilitate comparison, one specimen was tested for 5 yield points, and the other was tested for 7 yield points. The test results are shown in Figure 9, Figure 10 and Figure 11(“xx#”is a specimen mark). The test process runs from 5 to 1 and from 7 to 1 (see the figure for details).

As can be seen in Figure 9, Figure 10 and Figure 11, no matter what kind of pre-deformation is applied, the difference in the number of measured points can be observed (1) when the test order is the same (counterclockwise or clockwise order), but the test yield points are not the same, and the measured yield surfaces are very different. The first measured yield point is very close, and the difference between the following measured points increases obviously; (2) The size of the yield surface with 7 yield points is obviously larger than the size of the yield surface with 5 yield points, and the phenomenon of small pre deformation is more obvious (see Figure 11). This phenomenon is more pronounced in the case of small deformation. The above results show that the differences in the experimental results of the subsequent yield surface measured by 5 and 7 test points under 3 pre-deformation modes are consistent. It seems that it can be concluded that these differences are not caused by individual differences among specimens. The reason is that the subsequent observation points measured using a single sample are affected by accumulated residual strain at the previous measurement points. The result implies that the more the measured points for a single sample, the greater the deviation from the true yield surface will be. That is, it is impossible for single-sample method to adequately describe the subsequent yield surface with sufficient data points.

### 4.2. Discussion of Differences in Test Results Due to Different Test Sequences

**Figure 12:**
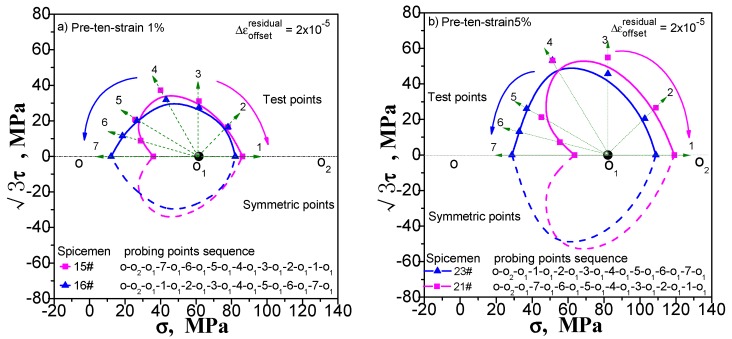
Influence of test point sequence on subsequent yield surface measured by single-sample method: (**a**) probing after pre-tension at 1%; (**b**) probing after pre-tension at 5%.

**Figure 13:**
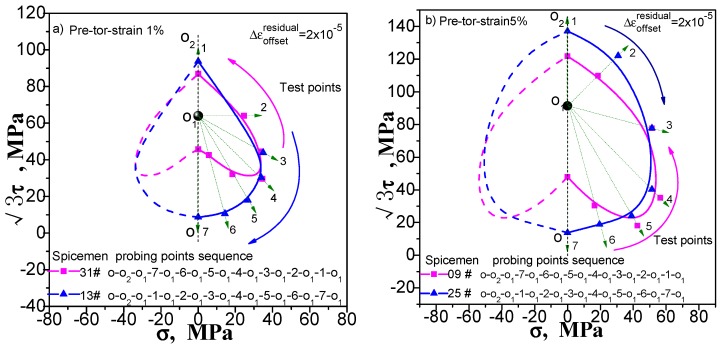
Influence of test point sequence on subsequent yield surface measured by single-sample method: (**a**) probing after pre-torsion at 1%; (**b**) probing after pre-torsion at 5%.

**Figure 14:**
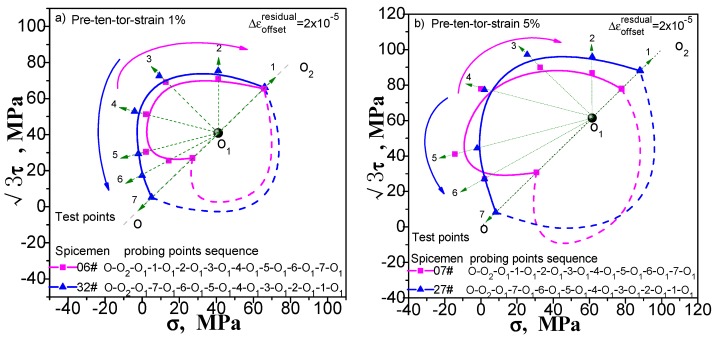
Influence of test point sequence on subsequent yield surface measured by single-sample method: (**a**) probing after combined pre-tension–torsion at 1%; (**b**) probing after combined pre-tension–torsion at 5%.

We still take the yield surface tests undergoing pre-tension at 1% and 5% pre-torsion at 1% and 5%, and combined pre-tension–torsion at 1% and 5% as an example to discuss the influence of different test sequences on the test results of the subsequent yield surface by comparing the test results under different test sequences. One test sequence is that after the pre-deformation unloading, the yield point in the opposite direction of the pre-deformation is measured first, and then the other yield points are measured in the specified order. The other test order is just the reverse: the yield point of the pre-deformation direction is measured first, and then the remaining yield points are tested in the opposite order. The results from the two test sequences vary widely, as shown in Figure 12, Figure 13 and Figure 14. The yield surface measured in the first order has an obvious concave in the opposite direction of the pre-deformation. The yield surface is tested in the second order not only does not appear concave, but also the yield point has a large bulge outward at the concave position measured in the first order.

The results suggest that, since affected by the cumulative plastic deformation on multipoint testing process, under the combined action of strain hardening and the Bauschinger effect, the single-sample method for yield surface test is sensitive to the number of test points, test paths and the test order. Different test scenarios lead to different test results, by which the reasonableness of the subsequent yield surface measured by the single-sample method is questionable.

## 5. The Test of Subsequent Yield Surface by Multiple-Sample Method

### 5.1. Test under Different Pre Deformation

**Figure 15:**
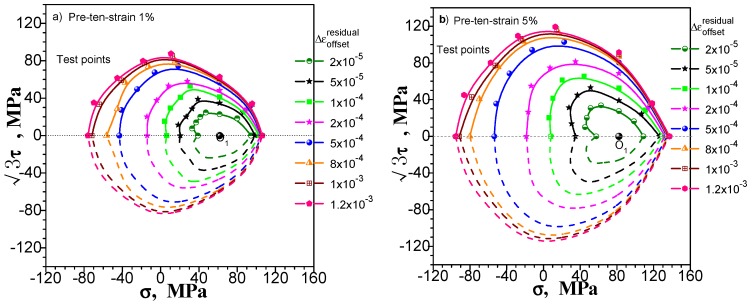
The subsequent yield surfaces measured by multi-sample method within different specified offset strain: (**a**) Probing after pre-tension at 1%; (**b**) probing after pre-tension at 5%.

**Figure 16:**
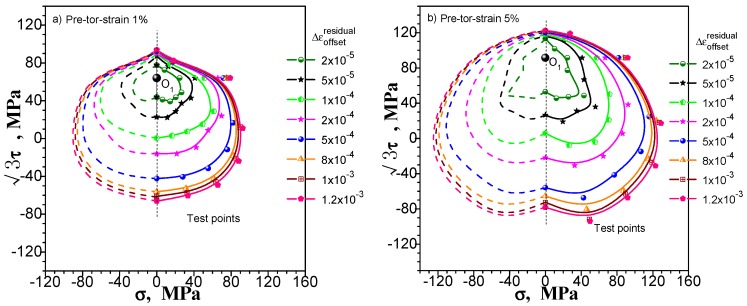
The subsequent yield surfaces measured by multi-sample method within different specified offset strain: (**a**) probing after pre-torsion at 1%; (**b**) probing after pre-torsion at 5%.

**Figure 17:**
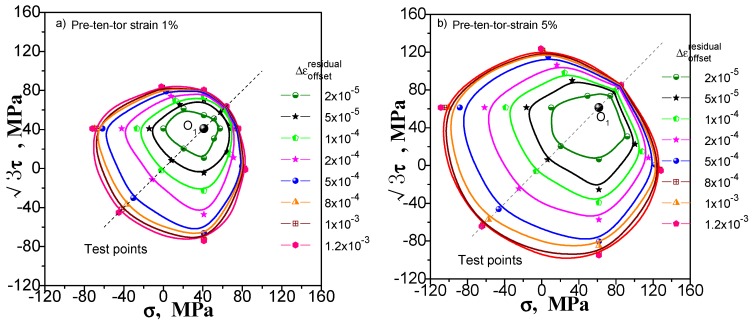
The subsequent yield surfaces measured by multi-sample method within different specified offset strain: (**a**) probing after combined pre-tension–torsion at 1%; (**b**) probing after combined pre-tension–torsion at 5%.

We still consider pre-tension at 1% and 5%, pre-torsion at 1% and 5%, and combined pre-tension–torsion at 1% and 5% for these three kinds of pre-deformation situation. Figure 15, Figure 16 and Figure 17 show the subsequent yield surfaces measured in accordance with the multiple-sample method at a specified offset strain of 20, 50, 100, 200, 500, 800, 1000 and 1200 με, respectively. The test results clearly reflect the following phenomena: (1) With the specified offset strain getting large, the size of measured yield surface increases (the yield point is farther from the unloading point), and the shape becomes more stable, even the results of pre tension-torsion deformations show good symmetry (Figure 17); (2) The effect of specified offset strain on the yield stress test is different in different directions: the direction of pre deformation is less affected by the change of offset strain. However, the curvature of the yield surface is considerably affected. The smaller the specified offset strain is, the more noticeable the sharp corner of the yield surface is.

**Figure 18:**
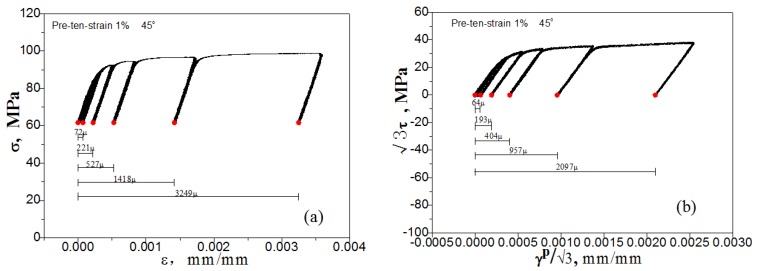
The relationship: (**a**) between axial stress and axial plastic strain; (**b**) between shear stress and shear plastic strain, in the direction of 45° during the yield test shown in Figure 15a.

From the above results, we can see that the multiple-sample method can measure the subsequent yield surface with a specified offset strain within a reasonable range. However, the single-sample method only achieves this level with difficulty. Because the test results of each test point of the multiple-sample method are independent of the others at different directions, so the test results are completely unaffected by the number of test points, the path and order, and the cumulative residual plastic strain of other points tested. Since the yield points of different offset strains can be determined in each direction, the multiple-sample method can be used to determine the subsequent yield surfaces under different offset strain. As an example, for the multiple-sample test of pre-tension at 1%, Figure 18 shows the axial stress, axial plastic strain, shear stress and shear plastic strain increasing/decreasing with loading/unloading, during the yield point test along the direction of 45° in the $\sigma \sim \sqrt{3}\tau$ plane which is shown in Figure 15a.

### 5.2. Discussion on the “Concave” Phenomenon of Measured Subsequent Yield Surface

In the prior section, the subsequent yield surface probed after combined pre-tension–torsion deformation is measured without the “concave” phenomenon (see Figure 17). The subsequent yield surfaces probed after pre-tension and pre-torsion deformation have obvious or distinguishable “concave” phenomena under the condition that the offset strain is not greater than 50 με (see Figure 15 and Figure 16). The subsequent yield surface probed after pre-tension deformation will have no “concave” phenomenon under the larger specified offset strain, but the subsequent yield surface probed after pre-torsion deformation at 5% still has the “concave” phenomenon (see Figure 16b)). In Section 2.1, for the single-sample method, both probed after pre-tension, pre-torsion (shear) or combined pre-tension–torsion deformation, the measured subsequent yield surfaces have the “concave” phenomenon in the opposite direction of the pre-deformation. However, in Section 4.2, when we reverse the test order, the measured subsequent yield surfaces after three kinds of pre-deformation will not appear as the “concave” phenomenon.

The test results of the subsequent yield surface in all cases mentioned above show that (1) whether the subsequent yield surface measured by the single-sample method has a definite concavity is related to the test order and may be significant or may not be present. Taking into account the rationality of the subsequent yield surface measured by the single-sample method, it is difficult to draw a conclusion on the basis of the single-sample method in view of the possibility of the “concave” phenomenon of the subsequent yield surface; (2) we have carried out six subsequent yield surface experiments under the smaller specified offset strain of multi-sample method, in which four kinds of experiments show different degrees of the “concave” phenomenon. However, only one of the subsequent yield surfaces experiments under the larger specified offset strain still has the “concave” phenomenon. Others show outward convexity. Taking into account the influence of the individual dispersion of the specimen on the multi-sample method, it is also assumed that the “concave” phenomenon of the individual yield surfaces is caused by the deviation of individual specimen. In a statistical sense, the measured subsequent yield surface can be assumed to be convex as long as the offset strain is large enough (for example, greater than 50 με).

**Figure 19:**
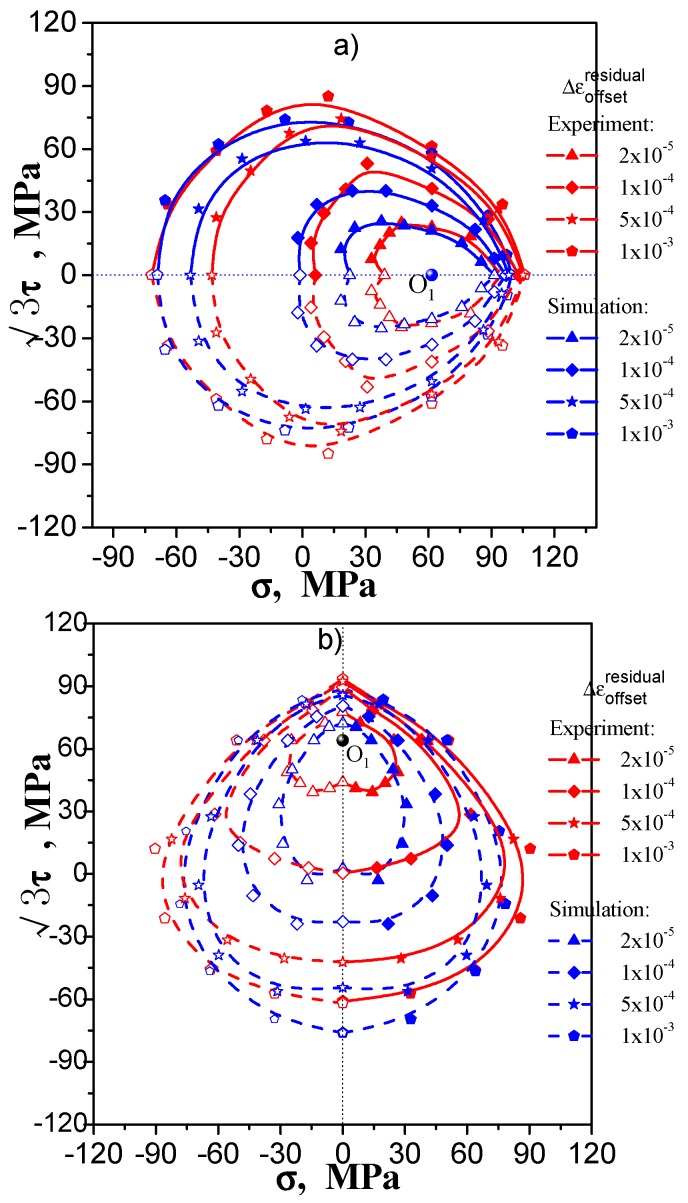
The test and simulated test subsequent yield surface by multiple-specimen method: (**a**) pre-tension at 1%; (**b**) pre-torsion at 1% [25].

It is difficult to give a definite explanation as to why the subsequent yield surface may be concave at a smaller specified offset strain, because the factors involved are very complicated. Since the deformation feature of the specimen’s structure, the strain of the outer wall of a thin-walled tubular specimen is larger than the inner wall’s, which may lead to a distinct difference between the inside and outside walls due to differences in grain orientation. Thus, the inhomogeneous deformation and residual deformation will result in fluctuations in the change of the Bauschinger effect occurring in different loading directions. The convexity of the yield surface discussed in plastic theory means the material is regarded as a continuous, consistent and non-porous medium. Whereas the actual metallic material has a complicated polycrystalline structure with large number of grains, its behavior is very different from the ideal continuum media. To study this issue further, the crystal plasticity simulation of the measuring process of the thin-walled sample, taking account of the polycrystalline deformation mechanism, may be applied. This has been conducted by Zhang et al. [25], from their simulations adopting multi-sample method by using thin-walled tube under combination of tension-torsion load, the measured subsequent yield surface may appear to be concave when yield point is determined by small offset strain (see Figure 19).

## 6. Conclusions

Using copper thin-walled tubular specimens, the subsequent yield surfaces respectively after pre-tension, pre-torsion and combined pre-tension–torsion are measured by employing the single-sample and multi-sample method, respectively; then, the rationality of the two testing methods is discussed and the following conclusions are obtained:

(1) Whether using the single- or multi-sample method, the measured subsequent yield surface is greatly different from the cylindrical yield surface proposed by the classical plastic theory.
(2) The shape and size of the subsequent yield surface measured by the single- and multi-sample method are also different. The accumulation of plastic deformation has an obvious influence on the subsequent yield surface measured by single sample method.
(3) Because the test results of each test point of the multiple-sample method are independent of the others at different directions, they are completely unaffected by the number of test points, the path and order, and the cumulative residual plastic strain of other points tested. Since the yield points of different offset strains can be determined in each direction, the multiple-sample method can be used to determine the subsequent yield surfaces under different offset strain.
(4) The subsequent yield surfaces may appear obviously concave measured both by the multi- and single-sample method. Whether the subsequent yield surface measured by the single-sample method has a definite concavity is related to the test order; whereas if the multiple-sample method is used, the concave phenomenon will only appear under the condition of small offset strain.
(5) Affected by the cumulative plastic deformation on multipoint testing process, the single-sample method yield surface test is sensitive to the number of test point, test path and the test order. Different test scenarios lead to different test results, and the reasonableness of the subsequent yield surface measured by the single-sample method is questionable.

## Figures and Tables

**Table 1:** Chemical composition in weight % of a T2 copper tube.

Chemical Composition/%						
Cu + Ag	Zn	Fe	S	Ni	Pb	impurity substance
≥99.9	≤0.005	≤0.005	≤0.002	≤0.005	≤0.005	≤0.1

**Table 2:** Mechanical properties of T2 pure copper.

E/GPa	G/GPa	$\sigma_{0.2}$/MPa	$\tau_{0.2}$/MPa	$\sigma_{u}$/MPa	$\tau_{u}$/MPa	$\varepsilon_{f}$
109.19	73.42	68.1	49.6	206.5	184.5	40.85%
